# Supplementary material for: Allylamine PECVD Modification of PDMS as Simple Method to Obtain Conductive Flexible Polypyrrole Thin Films
Source: Polymers (Basel). 2019 Dec 15;11(12):2108. doi: 10.3390/polym11122108 (PMC6960888; doi:10.3390/polym11122108)
Supplement: Supplementary file 1 [file polymers-11-02108-s001.pdf]

Allylamine PECVD modification of PDMS as simple method to obtain conductive flexible polypyrrole thin films.

Robert Texidó and Salvador Borrós *

[1] Grup d'Enginyeria de Materials (GEMAT), Institut Químic de Sarrià, Universitat Ramon Llull, Via Augusta, 390, 08017 Barcelona, Spain; roberttexidob@iqs.url.edu (R.T.)

* Correspondence: salvador.borros@iqs.edu

Table of Contents

I. Plasma reactor used to perform allylamine grafting 2

II. Deposition of PPy:PSS nanosuspensions on PDMS substrates without modification 3

III. Roughness profile of PPy deposited on Allylamine modified PDMS 3

I. Plasma reactor used to perform allylamine grafting

This reactor consists on a stainless-steel chamber (diameter, 25.5 cm; length, 41.6 cm) vertical plate reactor. The ground electrode is the reactor chamber, and the radio frequency (RF) electrode is an aluminum plate, which is used to hold the samples for polymerization. Additionally, the RF electrode is connected to a RF pulse generator (13.56 MHz) via a matching box. Gases and monomers are supplied via a standard manifold with gas fluxes adjusted with a tree of needle valves. The system pressure is monitored using a vacuum gauge controller (MKS PDR900, Andover, MA, USA) connected with a cold cathode/micropirani vacuum transducer (MKS 972 DualMag) positioned at the centre of the reactor. The system has a CO_2 / acetone cooled trap and a chemical trap filled with active carbon connected to avoid nonreacted monomer from reaching the pump (Trivac D 16BCS/PFPE Leybold, Cologne, Germany).

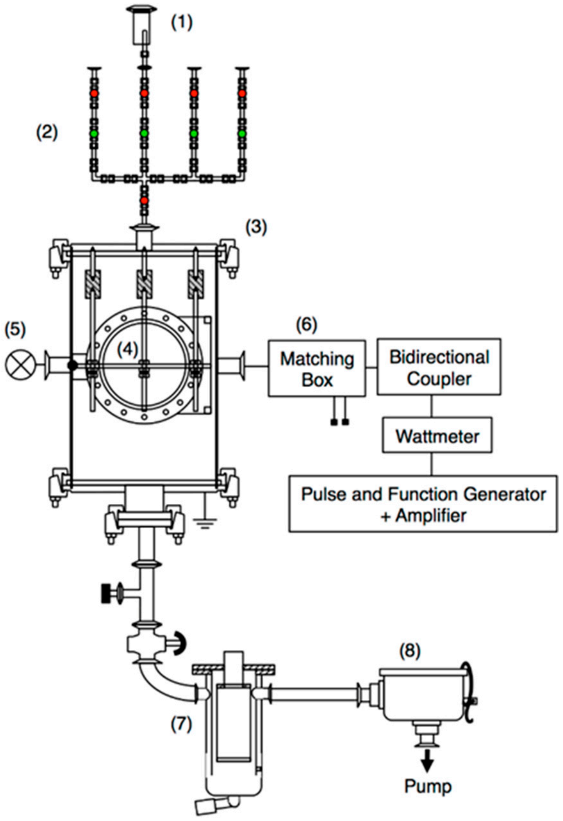

Fig. S1: Schematic diagram of stainless steel vertical plasma reactor and its electrical components: (1) monomer feed, (2) gases feed, (3) cylindrical chamber, (4) holder sample, (5) pirani gauge, (6) matching box and electrical system, (7) cold trap, and (8) chemical trap. Image used with permission[37].

II. Deposition of PPy:PSS nanosuspensions on PDMS substrates without modification

PPy:PSS nanosuspensions described in 3.2 section were deposited on PDMS substrate directly by drop casting.

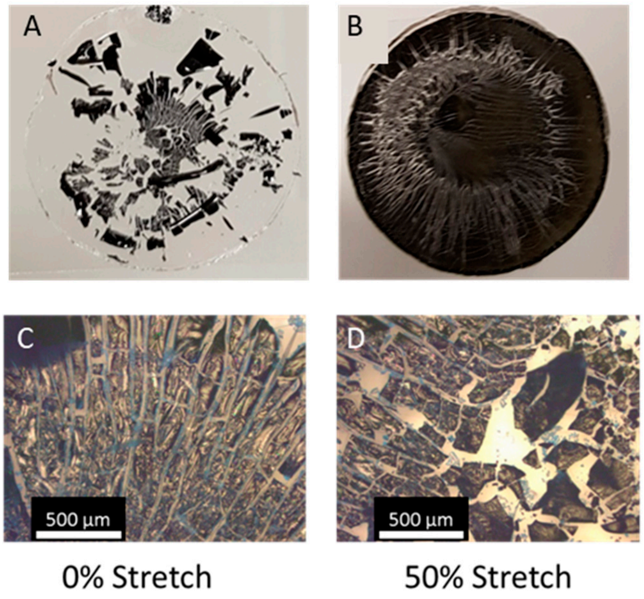

Fig. S2. PPy:PSS deposited on PDMS substrates A) Directly on PDMS B) On PDMS after plasma activation (50W O_2 plasma activation) C) magnification of PPy:PSS deposited on PDMS after plasma activation D) magnification of PPy:PSS deposited on PDMS after plasma activation stretched.

III. Roughness profile of PPy deposited on Allylamine modified PDMS

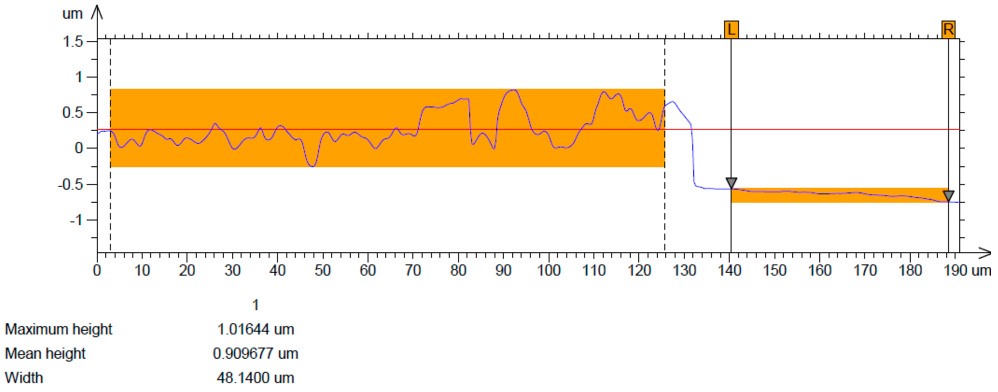

Fig. S3. Roughness profile of PPy deposited on allylamine modified PMDS measured through confocal microscopy.
